# Recombinant *Myxoma Virus*-Derived Immune Modulator M-T7 Accelerates Cutaneous Wound Healing and Improves Tissue Remodeling

**DOI:** 10.3390/pharmaceutics12111003

**Published:** 2020-10-22

**Authors:** Jordan R. Yaron, Liqiang Zhang, Qiuyun Guo, Enkidia A. Awo, Michelle Burgin, Lauren N. Schutz, Nathan Zhang, Jacquelyn Kilbourne, Juliane Daggett-Vondras, Kenneth M. Lowe, Alexandra R. Lucas

**Affiliations:** 1 Center for Personalized Diagnostics and Center for Immunotherapy, Vaccines and Virotherapy, The Biodesign Institute, Arizona State University, Tempe, AZ 85287, USA; jyaron@asu.edu (J.R.Y.); qguo27@asu.edu (Q.G.); eawo1@asu.edu (E.A.A.); mburgin@asu.edu (M.B.); lschutz2@asu.edu (L.N.S.); nzhang55@asu.edu (N.Z.); 2 Department of Animal Care and Technologies, Arizona State University, Tempe, AZ 85287, USA; jacki.kilbourne@asu.edu (J.K.); juliane.daggett@asu.edu (J.D.-V.); kenneth.m.lowe@asu.edu (K.M.L.)

**Keywords:** recombinant protein therapeutic, wound healing, chemokine, tissue remodeling, immune modulator

## Abstract

Complex dermal wounds represent major medical and financial burdens, especially in the context of comorbidities such as diabetes, infection and advanced age. New approaches to accelerate and improve, or “fine tune” the healing process, so as to improve the quality of cutaneous wound healing and management, are the focus of intense investigation. Here, we investigate the topical application of a recombinant immune modulating protein which inhibits the interactions of chemokines with glycosaminoglycans, reducing damaging or excess inflammation responses in a splinted full-thickness excisional wound model in mice. M-T7 is a 37 kDa-secreted, virus-derived glycoprotein that has demonstrated therapeutic efficacy in numerous animal models of inflammatory immunopathology. Topical treatment with recombinant M-T7 significantly accelerated wound healing when compared to saline treatment alone. Healed wounds exhibited properties of improved tissue remodeling, as determined by collagen maturation. M-T7 treatment accelerated the rate of peri-wound angiogenesis in the healing wounds with increased levels of TNF, VEGF and CD31. The immune cell response after M-T7 treatment was associated with a retention of CCL2 levels, and increased abundances of arginase-1-expressing M2 macrophages and CD4 T cells. Thus, topical treatment with recombinant M-T7 promotes a pro-resolution environment in healing wounds, and has potential as a novel treatment approach for cutaneous tissue repair.

## 1. Introduction

Dermal wounds, especially those complicated by factors such as diabetes, infection and age, represent a major medical burden, estimated to account for an annual expenditure of more than USD 20 billion by 2024 [1]. New approaches to manage and improve the healing of cutaneous wounds are the focus of intense investigation. As the first barrier protecting the host from external insults, the skin contains an intricate immune system that rapidly responds to limit infection, to remove debris and to repair damage. Cutaneous wound healing is a complex multi-stage process that relies upon numerous cell types and mediators acting in a coordinated temporal sequence. The wound healing process is commonly described in four phases: (i) hemostasis (ii) inflammation, (iii) tissue generation (proliferation) and (iv) remodeling [2]. Dysregulation at any stage of wound healing prolongs the resolution process, worsens scarring and leads to tissue disruption and the risk of infection. The immune system plays a critical role in successful wound healing. In addition to contributing to host defenses against infection, immune cells are critical regulators of wound healing through the secretion of cytokines, chemokines and growth factors that orchestrate local inflammatory cell invasion and responses, cellular differentiation, and tissue regeneration [3,4]. The immune cells involved in wound healing include all classes of neutrophils, macrophages, and T and B lymphocytes. An imbalance of immune cell function or discordance in cell orchestration at any stage can result in impaired wound healing. Tools to “fine tune” the immune response my lead to better treatment management and improve wound healing.

Chemokines are key players in the cellular orchestration that regulates wound healing. While they are involved in all stages of wound healing, chemokines are most abundant and varied during the inflammation and proliferation stages [5]. Chemokines recruit leukocytes, stimulate the activity of neutrophils, drive macrophage activity and polarization, and regulate the proliferation of fibroblasts and keratinocytes, which mediate collagen deposition [6]. Chemokines are also closely involved in angiogenesis, new vessel growth and extension [6]. There are approximately 20 chemokines that are known to be involved in wound healing, from across all four chemokine subfamilies (C, CC, CXC, and CX3C). Chemokines released from injured tissues bind to glycosaminoglycans (GAGs) on the surfaces of cells, in the vascular lumen glycocalyx or within extracellular matrices, where they may signal directly to cells, stimulating inter- and intra-cellular cascades, or drive chemotactic migration towards the site of injury [7]. Based on the understanding of the structure and function of these chemokines, research interest has intensified around the development of new therapeutic methods to accelerate wound healing, or to repair wounds with impaired healing by chemokine modulation [8,9]. These methods include chemokine depletion with biomaterials containing GAGs [10], immunotherapy with monoclonal antibodies against individual chemokines and chemokine receptors [9], or the topical application of recombinant chemokines [11]. While the basis of some of these approaches remains incompletely understood, each has demonstrated success in preclinical experiments.

Our group and others have investigated the application of immune modulators from viruses as an approach toward deriving novel protein therapeutics [12]. The co-evolution of viruses with their natural hosts invokes an adaptation arms race, whereby a successful strategy for the virus relies on immune evasion, often targeting key pathways that drive immune activation [13]. Large DNA viruses, such as poxviruses and herpesviruses, are adept at evading the innate immune system via a suite of virulence factors [14,15]. Translationally, these factors constitute a rich toolbox for developing immune modulators for treating disease [12].

Myxoma virus (MYXV) is a leporipoxvirus with well-known strict species-specificity and host-tropism to the European rabbit (*Oryctolagus cuniculus*) [16]. We have demonstrated the safety and immunotherapeutic efficacy of several MYXV immune modulators in a wide array of preclinical models [17,18,19,20,21,22,23,24,25,26,27]. M-T7 is an MYXV-derived immune modulator with broad chemokine-binding activity and proven therapeutic potential in inflammation-related diseases. M-T7 is expressed early in MYXV infection, and is the most abundantly secreted immune modulator [28]. In MYXV infection, M-T7 blocks lymphocyte infiltration into infected lesions by preventing chemokine gradient formation [29]. M-T7 is a soluble glycoprotein with the ability to directly bind all classes of chemokines (C, CC, CXC) tested in vitro, and decouple their interactions with GAG [30]. M-T7 treatment, given with or without concomitant cyclosporine, reduced acute renal transplant rejection, vasculopathy and scarring in rats [31]. M-T7 also markedly suppressed inflammatory cell invasion, and reduced acute and chronic aortic and renal transplant rejection, in mice in a manner dependent on heparan sulfation [23,32]. Thus, M-T7 has the potential to promote a resolution phenotype and a mechanism of tissue healing wherein the regulation of inflammation is critical. Here, we investigate the potential for recombinant M-T7 to modulate healing and local inflammatory cell responses at sites of full-thickness cutaneous wounds in a mouse model.

## 2. Materials and Methods

### 2.1. Recombinant M-T7 Production

Purified, recombinant M-T7 protein (m007L; NCBI Gene ID# 932081) was produced and provided by Viron Therapeutics (London, ON, Canada) and expressed and purified as previously described [33]. Briefly, the M-T7 coding sequence is inserted into pFastBacDual with a C-terminal His-tag and transformed into DH10Bac cells to generate bacmids as baculovirus shuttle vectors. Purified bacmids are transfected into Sf21 cells using Cellfectin II reagent. Supernatants containing baculovirus are used to transduce High Five cells. Secreted M-T7 is purified by affinity tag purification over a Ni-NTA column with further purification by size exclusion chromatography via FPLC with a HiLoad 16/60 Superdex 75 column.

### 2.2. Animals 

All animal procedures in this study were approved by the Institutional Animal Care and Use Committee of Arizona State University under protocol #17-1549R. Male and female wildtype C57BL6/J mice were bred on-site at Arizona State University. Mice aged 8–12 weeks were selected by simple randomization [34] and used in this study. Mice were kept on a standard 12 h light–12 h dark cycle in a specific pathogen-free environment and given food and water *ad libitum*. Mice were single-housed after the wounding procedure to prevent interference with wound healing, as previously described [35].

### 2.3. Wounding Surgery and Measurement

We performed a splinted, full-thickness wound healing model as previously described [35]. In this model, a silicone splint is used to prohibit the wound contraction of mouse skin around a single, intrascapular full-thickness biopsy punch wound during the first seven days, effectively forcing second-intention healing as occurs in human skin (whereas mouse skin primary heals by contraction).

Briefly, mice were anesthetized by intraperitoneal injection of 0.1 mL per 25 g bodyweight of a cocktail of 120 mg/kg ketamine and 6 mg/kg xylazine. Once reaching the anesthetic surgical plane (as determined by toe pinch), mice were prepped by shaving a 1x1 inch area spanning from between the ears to the apex of the spine and centered between each shoulder. The shaved area was sterilized by two successive washes of 2% chlorhexidine gluconate solution (Dyna-Hex 2^®^, Xttrium Laboratories, Prospect, IL, USA) followed by 70% ethanol with sterile cotton swabs. A small amount of veterinary ocular ointment was applied to each eye to prevent corneal drying. Mice were kept on a monitored heating pad for the duration of the procedure.

**Figure 1:**
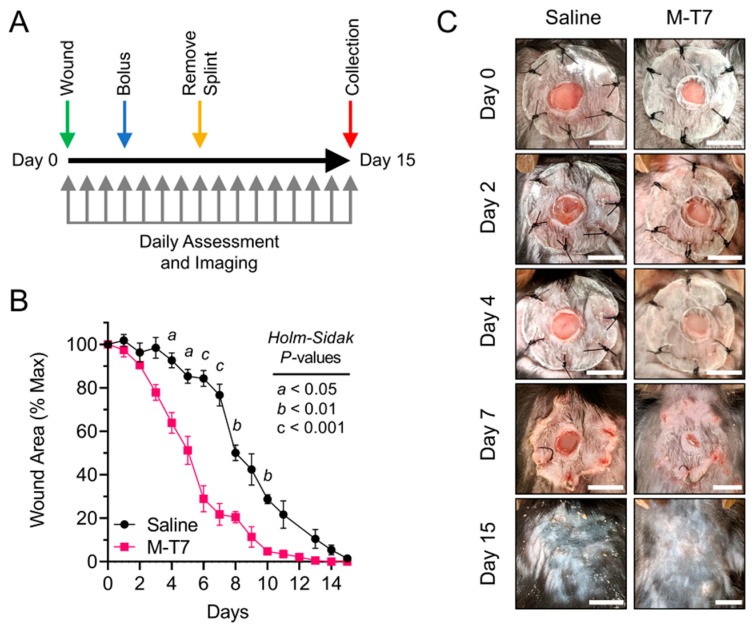
M-T7 accelerates full-thickness wound healing in mice. (**A**) Experimental design overview. Mice were wounded on day 0 (green arrow) and followed to day 15 post-wounding (red arrow), at which time mice were euthanized and tissue was collected. Mice were treated with saline or M-T7 on day 0 with a second bolus give on day 3 post-wounding (blue arrow). Mice were anesthetized and splints were removed on day 7 post-wounding (yellow arrow). Mice were assessed daily and images were collected for planimetric measurement of wound closure (gray arrows). *N* = 5 in each group. (**B**) Planimetric measurements of wound closure normalized to day 0 for each mouse. Mean and standard error are shown. Statistics were calculated by *T*-test per-day with correction for multiple comparisons by the Holm–Sidak method. For significance, *a* equates to *p* < 0.05, *b* equates to *p* < 0.01 and *c* equates to *p* < 0.001. (**C**) Representative wound images for saline and M-T7-treated mice on the day of wounding (day 0) and on days 2, 4, 7 and 15 post-wounding. The same mouse is shown in each image per condition. Scale bars are 5 mm.

A full-thickness excisional wound was created with a 3.5 mm biopsy punch tool centered in the shaved area. Careful attention was paid to prevent damage to the *panniculus carnosus* beneath the skin. Immediately after creating the punch, the wounds were treated by application of either 20 µL sterile normal 0.9% NaCl saline solution (*N* = 17) or 20 µL sterile normal saline (0.9% NaCl) containing 1 µg recombinant M-T7 (*N* = 16) applied directly to the wound bed with a micropipette. A donut-shaped silicon splint (O.D. 15 mm; I.D. 5.0 mm; Culture-Well™, Grace Biolabs, Bend, OR, USA) with Tegaderm™ (3M Company, Saint Paul, MN, USA) affixed to one side was coated with cyanoacrylate glue (Krazy Glue^®^) on the opposite side and carefully placed on the back of the mouse while keeping the wound centered within the inner diameter of the splint. Six interrupted sutures (4-0 black Ethilon monofilament with an FS-2 reverse cutting needle; Ethicon, Inc., Somerville, NJ, USA) were placed around the outer circumference of the splint (approximately 2 mm inset from the edge) to complete the procedure. Mice were monitored on heating pads until awake and motile prior to returning to single-housed cages for the remainder of the experiment. On day 3 post-wounding, the mice were anesthetized with 1–3% isoflurane, to effect, and 20 µL saline or 20 µL saline containing 1 µg M-T7 was carefully applied topically to the wounds (drop-wise above the wound) by inserting an insulin syringe through the silicon splint, with care not to disrupt the healing wound bed during application. To prevent self-induced secondary skin damage from scratching, mice were again anesthetized with 1–3% isoflurane, to effect, on day 7 post-wounding and the splints were carefully removed with sterile surgical scissors before being returned to single-housed cages as previously described [35]. The experimental design is outlined in Figure 1A.

### 2.4. Wound Planimetry

Mice were assessed while awake on the day of the procedure (day 0) and on every subsequent day of follow-up for a total of 15 days. Digital images were collected along with a known size marker. Planimetric measurements of the wound healing progress were performed in ImageJ/FIJI and calibrated against the known size marker [36].

### 2.5. Immunohistochemistry and Herocivi’s Polychrome Staining

Mice were euthanized in individual cohorts on days 2, 4, 7 and 15 post-wounding, and tissues were collected and fixed in 10% neutral-buffered formalin for 1 week before processing. Fixed tissues were processed and perfused with paraffin with a Leica TP1050 processor through graded alcohols and xylene, then embedded into paraffin cassettes on a Leica EG1160 embedding station. Blocks were sectioned using a Leica RM2165 microtome (5 µm sections) and stained with hematoxylin and eosin (H&E) according to standard procedures. Slides with sections that reached the wound site as determined by H&E screening were further stained by immunohistochemistry and collagen special staining.

Immunohistochemistry (IHC) was performed as previously described. Briefly, the slides were rehydrated through graded xylene and graded alcohols. Rehydrated slides were submerged in sodium citrate buffer, sandwiched with a clean glass slide to prevent tissue loss and incubated at 60 °C to retrieve epitopes. Endogenous peroxidases were quenched with 3% hydrogen peroxide in PBS and non-specific protein binding was blocked with 5% bovine serum albumin in TBS/0.1% Tween 20. Sections were probed overnight at 4 °C with rabbit polyclonal antibodies against Arginase-1 (Cell Signaling, #93668, 1:200), CD31 (Abcam, ab28364, 1:200), CD3 (Abcam, ab5690, 1:200) and CD4 (Abcam, ab183685, 1:1000), rabbit monoclonal antibodies against HSP47 (Abcam, ab109117, 1:300) or TGF-beta 1 (Abcam, ab215715, 1:500), or mouse monoclonal antibody against Ly6G (Invitrogen, #14-5931-82, 1:200). HRP-conjugated secondary antibodies against rabbit and mouse IgG (Jackson ImmunoResearch, West Grove, PA, USA) were applied at a dilution of 1:500 for 1 to 2 h at room temperature. Antigens were revealed with ImmPACT DAB (Vector Labs, Burlingame, CA, USA), counterstained with Gil’s formula #3 Hematoxylin and mounted with Cytoseal XYL.

Herovici’s Polychrome collagen stain kit was purchased from American MasterTech (Lodi, CA, USA). The slides were processed according to manufacturer’s procedure and mounted with Cytoseal XYL.

### 2.6. Histopathology Imaging and Analysis

The slides were assessed on an Olympus BX51 upright microscope equipped with an Olympus DP74 CMOS high-resolution camera operated by cellSens Dimensions v1.16. Objective-calibrated TIFFs were analyzed and processed in ImageJ/FIJI. Positively stained cells were quantified per high power field using the Cell Counter plugin developed by Kurt De Vos and packaged with FIJI under a GPLv3 license. Herovici’s Polychrome stains were quantified to produce a “Herovici Ratio” of pink stain (Type I collagen) versus blue stain (Type III collagen), where a higher ratio indicates more mature collagen and the less active deposition of immature collagen. Briefly, images were deconvoluted with the plugin *Colour Deconvolution 1.7* using the methods described by Ruifrok and Johnson [37]. A region of interest was drawn in the dermis of the wound area and replicated to both red and blue channels. The integrated intensity (densitometry) of the region of interest was measured for each channel and the values were used to produce the Herovici Ratio.

### 2.7. ELISAs

Enzyme-linked immunosorbent assays (ELISA) were performed using Duo-Set kits for TNFα (DY410), VEGF (DY493) and CCL2 (DY479), all from R&D Systems (Minneapolis, MN, USA). ELISAs were performed using tissues collected from individual cohorts of mice euthanized on days 1, 4 and 7. A 1 cm tissue sample centered on the wound was collected for each mouse, snap frozen and homogenized in RIPA lysis buffer according to manufacturer’s procedures. Results were normalized to mg total protein as determined by BCA protein assay (Pierce, ThermoFisher Scientific, Carlbad, CA, USA).

### 2.8. Statistics

Analysis of statistical significance was performed by Two-Way Analysis of Variance (ANOVA) and Student’s unpaired *T*-test using GraphPad Prism v8.2.1. All analyses passed normality tests according to Anderson–Darling (A2 *), D’Agostino–Pearson omnibus (K2), Shapiro–Wilk (W) and Kolmogorov–Smirnov (distance) with an alpha = 0.05. *P*-values were considered significant at * *p* < 0.05, ** *p* < 0.01, *** *p* < 0.001 and **** *p* < 0.0001, except in Figure 1B where *a* equates to *p* < 0.05, *b* equates to *p* < 0.01 and *c* equates to *p* < 0.001 for the purposes of clarity in data presentation.

## 3. Results

### 3.1. Recombinant M-T7 Promotes Full-Thickness Wound Healing

We analyzed the effects of recombinant M-T7 on the treatment of full-thickness wounds in a splinted wound healing model in wildtype C57BL6/J mice [35,38,39,40]. Based on effective doses and our prior work with a second unrelated MYXV-derived immune modulating serine proteinase inhibitor, Serp-1, in the same model, we gave recombinant M-T7 topically in a dose of 1 µg in 20 µL saline, with a second bolus of 1 µg in 20 µL saline 3 days post-wounding (Figure 1A) [35]. The control, saline-treated mice were similarly treated with a bolus of saline 3 days post-wounding. Daily planimetric measurements of wound healing progress demonstrate that M-T7 significantly accelerates full-thickness wound healing when given as a recombinant protein in a topical saline solution (Figure 1B,C). No evidence of wound site infection (pus, discharge, discoloration) was observed in either the saline or M-T7-treated groups. We noted that during the first 8 days of healing (day 0 and up to 7 days post-wounding), healing was limited to only second-intention mechanisms due to the silicon splint preventing wound contraction (Figure 1C). This is a major benefit of the silicon splint model because, left on its own, mouse skin contracts within 1–2 days, which limits the interpretation of the healing process. By day 7, saline control-treated wounds had only achieved a mean of 23% closure, while M-T7 significantly accelerated the healing process and a 78% mean wound closure was achieved (*p* = 0.0007). Even after removing the silicon splints, the wounds of M-T7-treated mice continued to close more quickly than mice treated with saline alone, and achieved full closure 3–4 days before saline-treated mice. Thus, recombinant M-T7 given during the early stage of healing has a sustained effect on accelerating wound closure.

### 3.2. Recombinant M-T7 Promotes Collagen Maturation in Wounds

**Figure 2:**
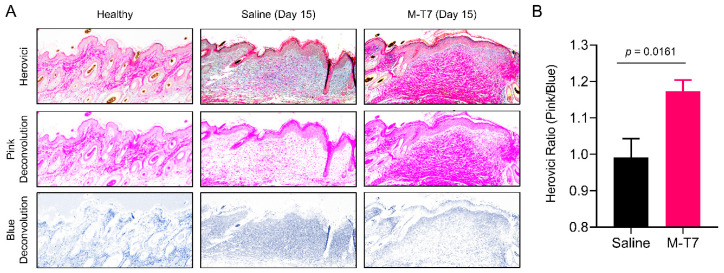
Quantitative assessment of collagen maturation in wounds treated with M-T7. (**A**) Representative micrographs of Herovici’s polychrome-stained normal skin and wounds at 15 days post-wounding. Top panels show brightfield data, while middle and bottom panels show color-deconvoluted fields for the pink and blue chromophores. (**B**) Quantification of collagen maturation in saline and M-T7 treated wounds by the Herovici Ratio, calculated by the densitometric ratio of the pink and blue chromophores in Herovici’s polychrome. Mean and standard error are shown. Statistics were calculated by *T*-test. *N* = 4 saline, *N* = 5 M-T7.

Collagen deposition and maturation are key components of the wound healing process. The inappropriate deposition or impaired maturation of collagen are associated with scarring and limited angiogenesis [41]. The improved remodeling of collagen in the healing wound can thus improve both scarring and healed tissue health via the promotion of improved angiogenesis. Herovici’s polychrome is a histologic special stain which differentiates between immature Type III collagen (stained blue) and mature Type I collagen (stained pink) [42]. We used Herovici’s polychrome to evaluate the collagen maturation of healed wounds after M-T7 treatment. Quantitative image analysis of the amount of pink Type I collagen staining versus the amount of blue Type III collagen showed what we refer to as the Herovici Ratio for the tissue. A higher Herovici Ratio (more pink, less blue) indicates more advanced collagen maturation, whereas a lower Herovici Ratio (less pink, more blue) indicates less collagen maturation and more active deposition of immature collagen. At day 15 post-wounding, the healed wounds of mice treated with M-T7 had significantly higher Herovici Ratios, indicating more mature Type I collagen than saline-treated mice (Figure 2). Interestingly, this was not associated with an increase in fibroblast marker HSP47 (Figure S1). These data indicate that, in addition to accelerated closure, wounds treated with M-T7 had a higher fidelity, healing with a more properly organized collagen architecture.

### 3.3. M-T7 Stimulates Peri-Wound Angiogenesis

**Figure 3:**
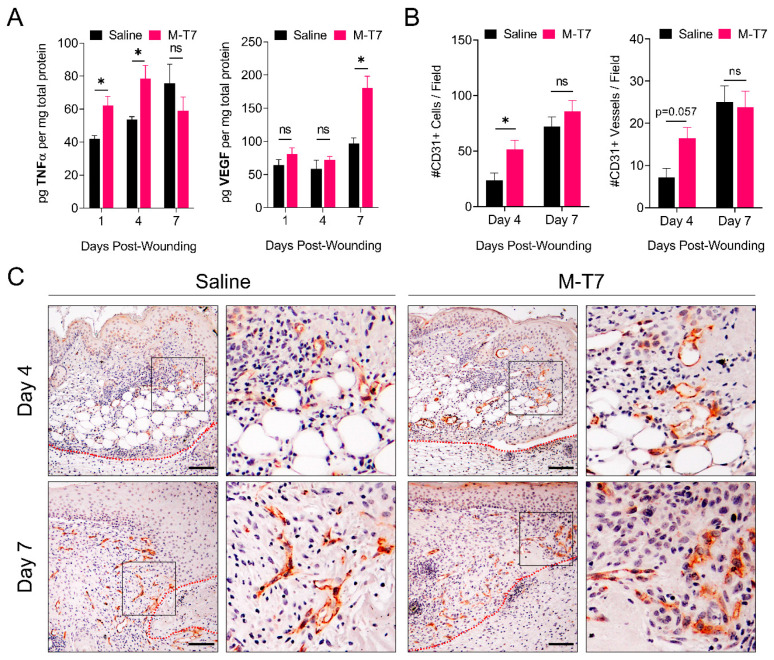
Assessment of peri-wound angiogenesis in wounds treated with M-T7. (**A**) ELISA quantification of TNFα and VEGF in wound tissues treated with saline or M-T7 collected on days 1, 4 and 7 post-wounding normalized to total protein. Bars are mean and standard error. Statistics were calculated by two-way ANOVA with Fisher’s LSD post-hoc analysis. (**B**) Quantification of CD31+ cells and vessels per 20× field in the peri-wound area of wounds treated with saline or M-T7 collected on days 4 and 7 post-wounding. Bars are mean and standard error. Two non-overlapping fields were quantified per mouse and statistics were performed on the average per mouse with the *N* = 4 per group. Statistics were calculated by two-way ANOVA with Fisher’s LSD post-hoc analysis. (**C**) Representative peri-wound CD31 IHC fields (10×) collected on days 4 and 7 post-wounding. Scale bars are 50 µm. Zoom areas indicated by boxes. *N* = 3–4 in each group and time point.

Angiogenesis is an essential component of the proliferative stage of cutaneous wound healing, characterized by an early and abundant burst of immature vessels which eventually regress into a mature vascular network via the activity of anti-angiogenic factors, such as Sprouty2 and PEDF [43]. Therapeutic strategies are now actively sought to enhance angiogenesis during wound healing [44]. Angiogenesis in the early stages of wound healing is driven by the priming of endothelial cells with pro-inflammatory cytokines, such as tumor necrosis factor alpha (TNFα), thereby inducing a tip cell phenotype [45]. In the context of tissue injury, TNFα is critical for the downstream production of vascular endothelial growth factor (VEGF), an essential growth factor in regulating angiogenesis [46]. We performed ELISA analyses of the healing wound’s bed tissue on days 1, 4 and 7 post-wounding to quantitatively measure levels of TNFα and VEGF (Figure 3A). Recombinant M-T7 induced a significant increase in wound bed levels of TNFα on days 1 and 4 (*p* < 0.05), and of VEGF by day 7 (*p* < 0.05), versus saline treatment alone. We performed immunohistochemistry of wound tissues on days 4 and 7 post-wounding to determine the degree of angiogenesis by staining for CD31 (also called PECAM-1), a canonical marker for endothelial cells in the vasculature (Figure 3B,C). A quantification of the number of CD31+ cells and vessels per 20× field in the peri-wound area indicated a significant increase on day 4 post-wounding (*p* < 0.05) versus saline treatment alone. We observed no significant difference on day 7 post-wounding. Qualitatively, we noted that the CD31+ cells formed more robust vessels in the wounds treated with M-T7, with increased length and thickness versus saline treatment alone (Figure 3C). Taken together, these results suggest that M-T7 stimulates an early TNFα response, which stimulates a more robust VEGF response, ultimately leading to accelerated angiogenesis in the peri-wound area associated with accelerated wound closure.

### 3.4. M-T7 Modulates Immune Responses in the Healing Wound

**Figure 4:**
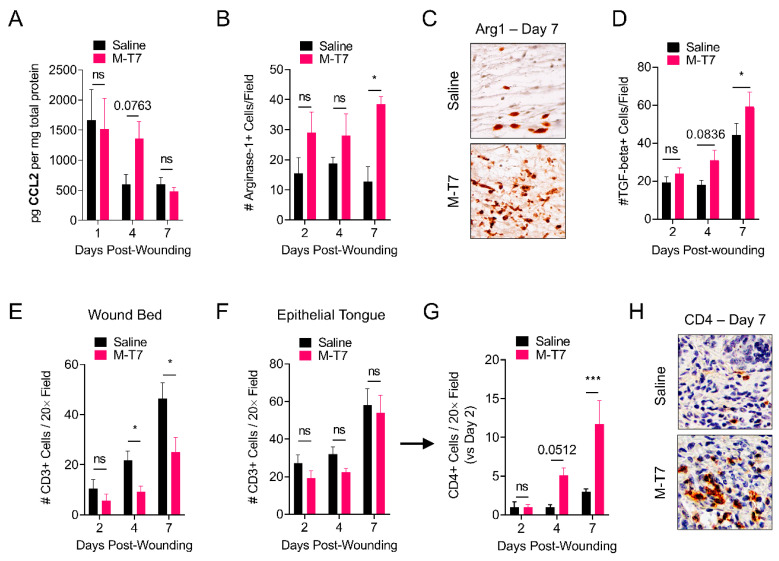
M-T7 modulates the immune response in the healing wound. (**A**) ELISA quantification of CCL2 in wounds treated with saline or M-T7 at days 1, 4 and 7 post-wounding, normalized to total protein. (**B**) Quantification of Arginase-1+ cells per 20× field of wounds treated with saline or M-T7 at days 2, 4 and 7 post-wounding. (**C**) Representative Arginase-1 IHC fields at day 7. (**D**) Quantification of TGF-beta+ cells per 20× field on days 2, 4 and 7 post-wounding. (**E**,**F**) Quantification of CD3+ cells per 20× field of wounds treated with saline or M-T7 at days 2, 4 and 7 post-wounding, specifically in the (**E**) wound bed or (**F**) epithelial tongue. (**G**) Quantification of CD4+ cells per 20× field of wounds treated with saline or M-T7 at days 2, 4 and 7 post-wounding normalized to the numbers on day 2. (**H**) Representative CD4 IHC fields in the epithelial tongue at day 7. Full 20× field is given in Figure S2. All bars are mean and standard error. Statistics are calculated by two-way ANOVA with Fisher’s LSD post-hoc analysis. *N* = 3–4 in each group and time point.

M-T7 binds to all classes of chemokines (C, CC and CXC) and inhibits their interaction with glycosaminoglycans, thereby preventing chemokine gradient formation [23,30,47]. CCL2, also called monocyte chemoattractant protein-1 (MCP-1), is a CC-class chemokine previously shown to have a critical role in the regulation of wound healing [48]. We performed the ELISA analysis of CCL2 on wound tissues treated with saline or M-T7, collected on days 1, 4 and 7 post-wounding (Figure 4A). Wounds treated with M-T7 had an elevated level of CCL2 on day 4 post-wounding, which approached significance (*p* = 0.0763), while levels of CCL2 were not different between saline and M-T7 treatment on days 1 and 7 post-wounding. Independent of its chemotactic function, the signaling of CCL2 with its receptor, CCR2, was previously shown to promote the polarization of macrophages towards a pro-resolution (i.e., M2) phenotype [49]. We performed immunohistochemistry of wounds treated with saline or M-T7 on days 2, 4 and 7 post-wounding, staining for Arginase-1, a canonical marker of M2 macrophage polarization. The quantification of Arginase-1+ cells revealed a trend towards elevated M2 macrophages on days 2 and 4 post-wounding, which achieved significance (*p* < 0.05) by day 7 post-wounding (Figure 4B,C). Accordingly, the number of TGF-beta+ cells per field trended towards significance on day 4 (*p* = 0.0836) and reached significance by day 7 post-wounding (*p* < 0.05) (Figure 4D). We further investigated the effects of M-T7 treatment on T cell infiltration in the healing wound. We found that M-T7 treatment significantly inhibited the infiltration of CD3+ T cells, a general T cell marker, into the bed of the healing wound on days 4 and 7 post-wounding (Figure 4E), without inhibiting the accumulation of CD3+ cells in the epithelial tongue of the wounds (Figure 4F). Regulatory T cells, a CD4 T cell subtype, are crucial for the normal and accelerated healing of cutaneous wounds [50]. We found that M-T7 treatment significantly increased the accumulation of CD4+ cells in the epithelial tongue of healing wounds versus saline treatment alone (Figure 4G,H, Figure S2). We did not observe an effect on neutrophil infiltration (Figure S3). Thus, decoupling the chemokine-glycosaminoglycan gradient with M-T7 modulates the immune response in the wound environment to accelerate healing.

## 4. Discussion

Large cutaneous wounds, particularly with associated poor healing (e.g., diabetic or aged), scarring and super-imposed infections, are a complex and costly medical burden, with an annual incidence of more than 6 million cutaneous wound cases and a collective yearly cost of more than USD 20 billion, not inclusive of the more than 170,000 scar revision surgeries annually in the United States [51]. Comorbidities such as advanced age and diabetes, or complications such as infection, burns and battlefield conditions, further increase the difficulty of wound management and the risk of adverse outcomes [52]. Investigation has thus intensified to address an unmet need for novel treatments to accelerate wound healing.

In this study, we tested the therapeutic efficacy of recombinant Myxoma virus-derived immune modulating protein M-T7 in a mouse model of full-thickness wound healing. We administered two doses of recombinant M-T7 on days 0 and 3 post-wounding, mirroring the dosing regimen that we found to be optimal for another Myxoma virus-derived immune modulator, Serp-1, in a previous study [35]. Planimetric analysis revealed a significant acceleration of wound closure by treating wounds topically with M-T7. Acceleration occurred during the earliest stages of healing and was independent of contraction, as the silicone splints were not removed until day 7 post-wounding (Figure 1B). This finding is promising, as the first phase of healing is known to be a crucial period for protection against infection and the prevention of additional trauma as granulation tissue is formed [53].

One potential risk of accelerated wound healing is the deposition of disorganized connective tissue leading to scarring, particularly in full-thickness skin wounds without contraction [54]. Druecke and colleagues investigated the use of different dermal regeneration templates on full-thickness wounds in a porcine model [55]. They found that while the Integra material, a composite scaffold of bovine hide collagen and shark chondroitin-6-sulfate, improved collagen maturation, there was slower tissue ingrowth, and tissue integrity was lost [55]. In contrast, the authors found that a bovine hide collagen sponge scaffold, produced via chemical crosslinking with 1-ethyl-3-(3-dimethylaminopropyl)carbodiimide (EDC), allowed more rapid ingrowth and overall tissue integrity, with no benefit to collagen maturation. Here, we found that in addition to accelerating wound healing, recombinant M-T7 also resulted in earlier, improved collagen maturation, as determined by quantification with Herovici’s polychrome in a metric we term the “Herovici Ratio” (Figure 2). A similar quantification of Type III:I collagen was observed by O’Rourke and colleagues in their investigation of accelerated wound healing by surfactant polymer dressings containing siRNA to Fidgetin-Like 2, with the authors noting increased red Type I collagen, illustrating higher-fidelity healing [56].

Angiogenesis is a critical component of wound healing, and several comorbidities associated with impaired wound healing, such as diabetes, also exhibit reduced angiogenesis [57,58]. Angiogenesis plays a critical role in providing the sufficient delivery of blood and nutrients, and access to reparative immune cells, during the course of healing. Accordingly, numerous groups have investigated approaches to accelerate healing by promoting angiogenesis. For example, recombinant TNFα applied directly to wounds accelerates the early stages of wound healing [59]. The therapeutic effect of TNFα in the early stage of wound healing may proceed via the induction of angiogenesis, as TNFα is a powerful inducer of VEGF and endothelial tip cell formation [45,46]. Other treatments shown to accelerate wound healing also act via the induction of angiogenesis. For example, topical simvastatin and asperosaponin VI both act to accelerate wound healing by enlisting the VEGF signaling cascade, and VEGF-C applied directly to wounds also accelerates healing [60,61,62]. Here, we show that a recombinant M-T7 treatment resulted in a significant increase in local TNFα during the earliest stages of wound healing, which temporally transitions into a significant increase in VEGF in the healing bed (Figure 3A). This coincides with the canonical, early inflammatory phase of healing, and the transition to the proliferation phase of healing [63]. Accordingly, increased angiogenesis was directly observed by immunohistochemistry for CD31 in the peri-wound area (Figure 3B,C). Thus, the data suggest that topical M-T7 modulates the chemokine environment, resulting in the augmentation of pro-healing molecules in the wound environment, engaging the pro-angiogenesis signaling cascade at the level of both cytokines and growth factors, and resulting in a significant induction of angiogenesis at the boundaries of healing wounds associated with increased wound closure.

We sought to determine the effect of recombinant M-T7 on local immune responses in the healing wounds. Some virus-derived immune modulators, such as the herpesvirus M3 chemokine decoy receptor and Myxomavirus MT1, inhibit the ability of chemokines to signal to their receptor. M3 also uniquely blocks chemokine binding, both to GAGs and also to receptors [64]. In contrast, M-T7 acts primarily at the level of chemokine-glycosaminoglycan interactions [30,47,65,66]. M-T7 is an interferon gamma receptor homologue with specificity for rabbit interferon gamma [28]. Interestingly, the M-T7 inhibition of chemokine to GAG binding is found for all mammals tested to date, e.g., rabbits, rats, mice and human cells [30]. Thus, M-T7 treatment is expected to inhibit chemokine gradient formation [47]. Indeed, we previously found that M-T7 lost therapeutic efficacy in the absence of normal active heparan sulfation in mice with conditional endothelial deficiency of the heparan sulfotransferase enzyme Ndst1, with presumed consequences in modifying the formation of chemokine gradients [23]. CCL2/MCP-1 signaling is known to be critical in regulating physiologic wound healing. Low and colleagues reported that wounds made in mice deficient of CCL2 exhibit delayed re-epithelialization, reduced capillary density and impaired collagen remodeling [48]. In contrast, multiple groups have shown that recombinant CCL2 treatment reverses impaired wound healing in diabetic mice by restoring macrophage responses [67,68]. Receptor engagement by chemokines induces the internalization of both the receptor and its ligand, resulting in intracellular degradation and recycling [69]. While CCL2 exists in dynamic equilibrium as both a monomer and dimer, only the monomeric form is capable of receptor engagement, and obligate dimeric mutants of CCL2 are incapable of signaling [70]. Importantly, the dimerization of CCL2 requires glycosaminoglycan interactions [71]. We found increased levels of CCL2 in healing wounds when treated with recombinant M-T7 (Figure 4A), consistent with the role of CCL2 in improved healing. We hypothesize that M-T7 treatment inhibited the oligomerization of CCL2, slowing receptor engagement and delaying its subsequent degradation. Investigating further, we found M-T7-dependent effects in two cell populations known to be affected by CCL2 and other chemokine signaling: macrophages and T cells. Specifically, we found an increase in M2-polarized, pro-resolution macrophages (Figure 4B). This finding agrees with prior work showing that CCL2 signaling results in M2 polarization [49]. We also found increased CD4+ T cells in the epithelial tongues of healing wounds treated with M-T7 (Figure 4F,G), in agreement with the ability for CCL2 to promote CD4 recruitment, and in the CD4-lineage cells driving accelerated wound healing [50,72]. CCL2 acts directly on T cells via the action of CCR2 and CCR4 [73], but can also induce the recruitment of CD4 cells into tissues in a promiscuous manner, using other receptors [72]. Further, M-T7 interacts with many chemokines, and may induce a broad milieu change in a range of chemokines. It will be important to probe the specific targeted mechanisms of CD4 recruitment using genetic knockouts or neutralizing antibody treatments in future studies. We speculate that the continued expression of CCL2 by local cells, combined with an off-rate of CCL2:M-T7 interactions, may have contributed to sustained CCL2 signaling. However, we cannot exclude the role of other chemokines or cytokines in the wound healing milieu in this study, as M-T7 interferes with GAG binding for C, CC and CXC chemokines in vitro. Thus, the decoupling of glycosaminoglycan interactions with chemokines by M-T7 has an effect on CCL2 signaling, and downstream effects on macrophage and T cell populations, leading to accelerated wound healing. Further work is necessary to determine the specific chemokine and GAG pathways modulated by M-T7 in the orchestration of local and infiltrating immune cells in the healing wound bed.

M-T7 is now the second Myxoma virus-derived immune modulator to exhibit efficacy in promoting wound healing. Serp-1, a serine protease inhibitor (serpin), is a glycosylated, secreted protein which targets serine proteases in both the thrombotic (FXa, thrombin) and thrombolytic (uPA, tPA, plasmin) cascades [20]. We recently reported that recombinant Serp-1 accelerated full-thickness wound healing in mice, with numerous observed similarities to the M-T7 treatment as performed in this study [35]. In addition to the acceleration of wound closure, both Serp-1 and M-T7 resulted in an increase in VEGF and peri-wound angiogenesis, as well as an increase in pro-resolution M2-polarized macrophages. These similarities underscore the benefits associated with developing therapeutics from virus-expressed immune modulators. These studies also again emphasize the safety of these immune modulators when used as therapeutics. First, the limited genomic space in a virus necessitates the evolution of multipotency (i.e., multiple targets of inhibition), providing highly potent and effective immune modulating molecules [74]. While Serp-1 targets numerous proteins in the clotting cascade, M-T7 targets the panoply of chemokines. Second, virus-derived immune modulators often exhibit potency at extremely low concentrations. Both Serp-1 and M-T7 function at doses of only 100 nanogram per gram bodyweight, the equivalent of microgram per kilogram dosing in humans; that is, the lowest end range for therapeutic biologics [75]. Indeed, Serp-1 has been shown to exhibit therapeutic efficacy down to the picogram per gram range [76]. Third, virus-derived immune modulators have undergone extensive “research and development” in the evolutionary arms race between the virus and its host. In the case of Myxoma virus, an estimated 10 million years of evolution have gone into developing expert modulators of the host immune response [16]. Thus, the suite of immune modulating proteins in Myxoma virus are a valuable, highly optimized “medicine cabinet” for targeting immune-driven pathologies, and for harnessing immune function to enhance tissue repair [12]. Further work will investigate the potential for combining these proteins in cocktails, towards further enhancing therapeutic responses in wound healing and other models.

## 5. Conclusions

We report here that treatment with recombinant M-T7, a Myxoma virus-derived chemokine signaling modulator, accelerates the rate of healing in full-thickness wounds in wildtype mice. M-T7 treatment improved connective tissue remodeling, and increased angiogenesis and pro-resolution immune cell phenotypes. The chemokine milieu of the healing wound bed is highly complex, and M-T7 can interact with all classes of the C, CC and CXC chemokines. We observed an effect on CCL2 in the present study, which was associated with effects on macrophages, T cells and endothelial cells. Further work is needed to delineate the precise mechanisms of M-T7’s therapeutic effects on wound healing. Further, it will be important to investigate the effects of recombinant M-T7 in complex comorbidities such as diabetes, infection and burns, to develop next-generation versions of M-T7 with enhanced function, and to learn more about the fundamental role of chemokines in cutaneous wound healing. Thus, M-T7 represents a promising virus-derived therapeutic, a new class of protein biologics with the potential to address the significant medical burden created by dermal wounds.

## Supplementary Materials

The following are available online at https://www.mdpi.com/1999-4923/12/11/1003/s1, Figure S1: Quantification of IHC staining for HSP47+ cells per 20× field on tissues of mice on days 4 and 7 post-wounding and treated with saline or M-T7. Statistics analyzed by two-way ANOVA with Fisher’s LSD post-hoc analysis. All bars are mean and standard error. *N* = 3–4 mice per treatment per time point, Figure S2: Full-frame 20× fields of CD4 IHC on Day 7 post-wounding for mice treated with saline and M-T7. Corresponds to Figure 4H in the main manuscript. Images are representative of 3–4 mice per treatment, Figure S3: Quantification of IHC staining for Ly6G+ cells per 20× field on tissues of mice on days 4 and 7 post-wounding and treated with saline or M-T7. Statistics analyzed by two-way ANOVA with Fisher’s LSD post-hoc analysis.
