# Supplementary material for: Recombinant Myxoma Virus-Derived Immune Modulator M-T7 Accelerates Cutaneous Wound Healing and Improves Tissue Remodeling
Source: Pharmaceutics. 2020 Oct 22;12(11):1003. doi: 10.3390/pharmaceutics12111003 (PMC7690590; doi:10.3390/pharmaceutics12111003)
Supplement: Supplementary file 1 [file pharmaceutics-12-01003-s001.pdf]

pharmaceutics

MDPI

Supplementary Materials: Recombinant *Myxoma Virus*-Derived Immune Modulator M-T7 Accelerates Cutaneous Wound Healing and Improves Tissue Remodeling

Jordan R. Yaron, Liqiang Zhang, Qiuyun Guo, Enkidia A. Awo, Michelle Burgin, Lauren N. Schutz, Nathan Zhang, Jacquelyn Kilbourne, Juliane Daggett-Vondras, Kenneth M. Lowe and Alexandra R. Lucas

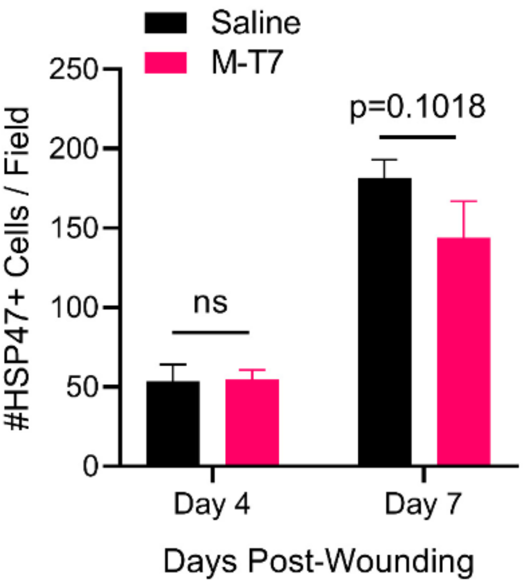

Figure S1. Quantification of IHC staining for HSP47+ cells per 20× field on tissues of mice on days 4 and 7 post-wounding and treated with saline or M-T7. Statistics analyzed by two-way ANOVA with Fischer's LSD post-hoc analysis. All bars are mean and standard error. N = 3-4 mice per treatment per time point.

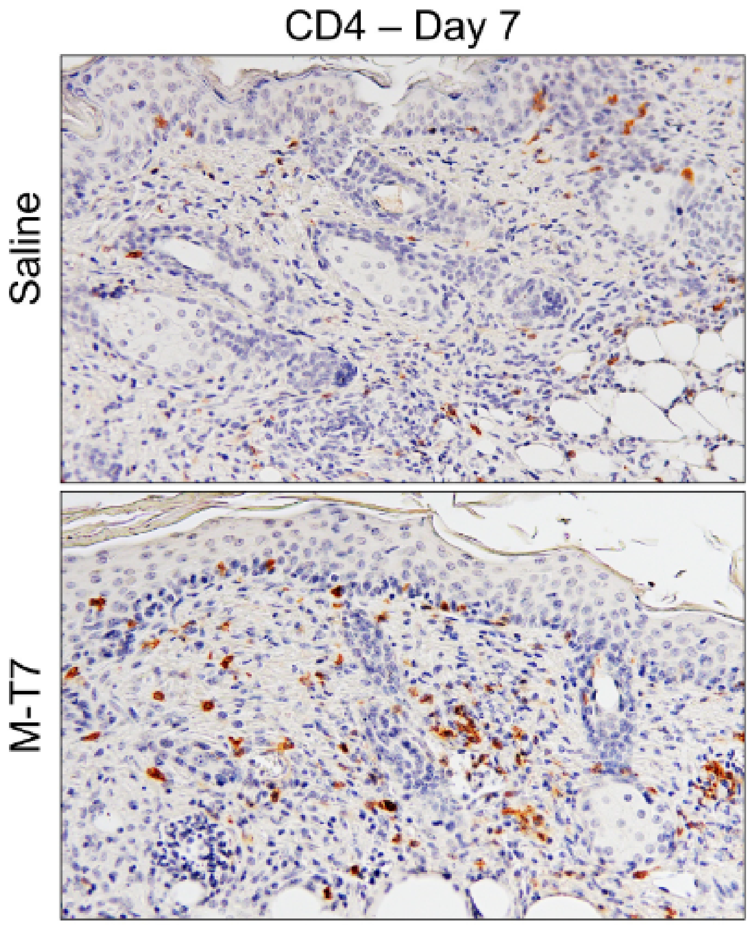

Figure S2. Full-frame 20× fields of CD4 IHC on Day 7 post-wounding for mice treated with saline and M-T7. Corresponds to Figure 4H in the main manuscript. Images are representative of 3–4 mice per treatment.

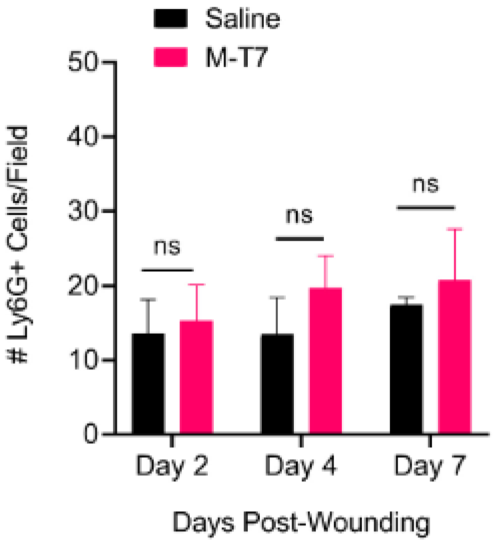

Figure S3. Quantification of IHC staining for Ly6G+ cells per 20× field on tissues of mice on days 4 and 7 post-wounding and treated with saline or M-T7. Statistics analyzed by two-way ANOVA with Fischer's LSD post-ho.
